# The FGL-1/LAG-3 Axis is Associated With Disease Course in Alcohol-associated Hepatitis: A Preliminary Report

**DOI:** 10.1016/j.jceh.2024.102424

**Published:** 2024-10-10

**Authors:** Lasse Pedersen, Lotte L. Eriksen, Frederik H. Brix, Hendrik Vilstrup, Bent Deleuran, Thomas D. Sandahl, Sidsel Støy

**Affiliations:** ∗ Department of Hepatology and Gastroenterology, Aarhus University Hospital, Aarhus, Denmark; † Department of Clinical Medicine, Aarhus University, Aarhus, Denmark; ‡ Department of Rheumatology, Biomedicine, Aarhus University Hospital, Aarhus, Denmark; § Department of Biomedicine, Aarhus University, Aarhus, Denmark

**Keywords:** fibrinogen-like protein 1, lymphocyte activation gene 3, alcohol-associated liver disease, acute phase protein, checkpoint inhibitor

## Abstract

**Background:**

Alcohol-associated hepatitis (AH) has a short-term mortality rate of up to 40% primarily related to impaired hepatocyte regeneration and uncontrolled liver inflammation. The acute phase protein fibrinogen-like protein 1 (FGL-1) produced by hepatocytes stimulates hepatocyte proliferation by autocrine signaling. FGL-1 also is a ligand for the inhibitory T cell receptor lymphocyte activation gene 3 (LAG-3). In these ways, FGL-1 and LAG-3 have beneficial interactions that could be interrupted in AH.

**Aims:**

We aimed to characterize FGL-1 and LAG-3 in patients with AH and describe their relationship with the disease state and course.

**Methods:**

Thirty-two patients with AH were included at diagnosis and followed up for 3 years. We measured the hepatic gene expression of FGL-1 and LAG-3 using RNA sequencing, plasma FGL-1 and soluble (s)LAG-3 using ELISA, and LAG-3^+^CD8^+^ T cells using flow cytometry. Healthy persons (HC) and patients with stable alcohol-associated cirrhosis served as controls.

**Results:**

At diagnosis of AH, liver FGL-1 mRNA was increased when compared to HC, whereas plasma FGL-1 was unchanged. In contrast, liver LAG-3 mRNA was reduced in AH. Plasma sLAG-3 levels and the frequency of LAG-3^+^CD8^+^ T cells were as in HC. However, those patients who had the lowest plasma FGL-1 and the lowest frequency of LAG-3^+^CD8^+^ T cells at diagnosis had the highest disease severity and mortality.

**Conclusions:**

Our data suggest that an impaired FGL-1/LAG-3 axis may be involved in the pathogenesis and course of AH.

Alcohol-associated hepatitis (AH) is a life-threatening disease with a six-month mortality rate of up to 40%.^1^ The incidence of AH is increasing; however, no pharmacological treatment has shown convincing effects on clinical outcomes. Therefore, the key treatment remains careful supportive care and moderate hyperalimentation.^2^^,^^3^ The disease mechanisms are only partially understood. Acute alcohol-induced liver damage results in uncontrolled inflammation of the liver, which involves both innate and adaptive immune cells, and this further exacerbates the liver injury. The avid inflammation is accompanied by defects in liver regeneration, and both problems together contribute to the high mortality from AH.^3^, ^4^, ^5^, ^6^, ^7^

Fibrinogen-like protein 1 (FGL-1) is an acute-phase protein produced by hepatocytes in response to interleukin (IL)-6, IL-22, and hepatocyte nuclear factor I.^8^^,^^9^ FGL-1 acts in an autocrine or paracrine manner to increase the proliferation of hepatocytes.^10^^,^^11^ In patients with AH, intact hepatocyte proliferation is strongly associated with positive outcome.^12^ Furthermore, a recent study showed that FGL-1 is a major ligand of Lymphocyte Activation Gene-3 (LAG-3).^13^ LAG-3 is an inhibitory receptor found primarily on activated CD4^+^, CD8^+^, and regulatory T cells (Treg), as well as on natural killer (NK) cells, and it binds the major histocompatibility complex class II (MHC-II).^14^, ^15^, ^16^ FGL-1 may bind to the LAG-3 receptor independently of MHC-II interaction, leading to decreased proliferation and cytokine production in T cells.^13^^,^^17^^,^^18^ Activity in the FGL-1/LAG-3 axis may, therefore, be of particular relevance for LAG-3 expressing CD8^+^ T cells.

The function and regulation of CD8^+^ T cells have previously been found to be altered in patients with alcohol-associated liver disease including AH^19^^,^^20^ Moreover, in AH CD8^+^ T cells exhibit elevated expression of other inhibitory receptors such as programmed death receptor (PD)-1 and T cell immunoglobin and mucin domain-containing protein (TIM)-3.^9^

We therefore hypothesized that FGL-1 and its anti-inflammatory receptor LAG-3 may be involved in the disease severity and course in patients with AH. Our aim was to study the expression of FGL-1 and LAG-3 in liver biopsies, the concentrations of plasma FGL-1 and soluble (s)LAG-3, and the frequency of LAG-3^+^ CD4^+^, and CD8^+^ T cells in patients with AH. We measured elevated liver FGL-1 expression and decreased liver LAG-3 expression. Furthermore, we found an association between relatively low plasma FGL-1, liver LAG-3, and frequency of LAG-3^+^ CD8^+^ T cells and more severe disease with adverse course.

## Methods

### Study Design and Population

Thirty-two patients diagnosed with AH were consecutively recruited from the Department of Hepatology and Gastroenterology, Aarhus University Hospital, Denmark from 2013 to 2017. The criteria for diagnosis were a history of excessive alcohol consumption (>50 g/day for men and >40 g/day for women), a period of abstinence of less than 4 weeks before disease presentation, presentation of acute jaundice within 2 weeks before diagnosis with serum bilirubin >80 μmol/L, and age between 18 and 75 years. The exclusion criteria were hepatocellular carcinoma, gallstones, other underlying liver diseases, upper gastrointestinal bleeding, uncontrolled infection, or immunomodulatory therapy within the previous 8 weeks. Upon admission, all patients were screened for infection, which included clinical examination, urine analysis, blood and ascites (if present) cultures, chest radiography, and other tests, if needed. Patients who suffered from an infection were treated with antibiotics and included when the infection was controlled. Diagnostic liver biopsies were performed in 24 patients by transjugular route.^21^ Local clinical guidelines at the time of sampling dictated a treatment regimen of either pentoxifylline (before 2015) or prednisolone when the Glasgow Alcoholic Hepatitis Score (GAHS) was 9 or above. Peripheral blood mononuclear cells (PBMC) and plasma samples were obtained from all patients on the day of diagnosis and 7 and 90 days after diagnosis. The patients were followed for 3 years. Follow-up blood samples were obtained from from 20 patients on day 7 and 12 patients on day 90, respectively. Blood samples from healthy controls (HC, n = 14, Female/Male = 7/7, mean age = 43 years, standard deviation = 10) and patients with stable alcohol-associated cirrhosis (n = 20) were obtained from the Department's Biobank and were used as controls. For RNA sequencing, liver tissue was obtained from the unaffected rim of livers resected for metastatic colon cancer (healthy tissue (HT), n = 11). The study was performed following the Declaration of Helsinki and written, informed consent was obtained before the inclusion of the patients. The study was approved by the Central Denmark Region Ethics Committee (no. 1-10-72-40-13), and Danish Data Protection Agency. This study was registered at clinicaltrials.gov (NCT01918462).

### RNA-sequencing of Liver Biopsies

Among the 24 patients who had diagnostic biopsies performed, we were able to secure an additional biopsy during the procedure from 14 patients. These biopsies were stored at −80 °C in RNALater (SakuraFinetek, US) until RNA extraction. The RNA was isolated using the NucleoSpin kit (MACHEREY-NAGEL, Düren, Germany), purified, and used to generate a copy DNA library using the NEBNext Ultra II Directional RNA Library Prep kit for Illumina (New England Biolabs, Ipswich, MA). A NextSeq 500 was then used with a NextSeq 500/550 High Output Kit V2 (Illumina, San Diego, CA, USA) to sequence the copy DNA libraries. These sequencing data were aligned to the human genome from the Ensembl database by Spliced Transcript Alignment to a Reference software (PMID:23104886).^21^ To normalize for differences in sequencing depth across biopsies, the R package DESeq2 version 1.18.1 was used. The differential expression analysis was performed on the complete dataset before the two genes reported here were extracted. Gene expression is reported as reads per kilobase million (RPKM) in patients with AH and patients undergoing liver resection (HT), and the log2 fold change is calculated with the HT group as a reference.

### Enzyme-linked Immunosorbent Assay (ELISA)

EDTA blood was centrifuged at 1800×*g* for 10 min at 4 °C to obtain plasma, which was stored at −80 °C until analysis. Optimized ELISAs were used to measure plasma FGL-1 concentration (BioSite, Denmark, cat. No. EKH6126), and sLAG-3 (Invitrogen, US, cat. no. BMS2211). All samples were analyzed in duplicate. Cut-off values were calculated as the average absorbance of the blank + 2 × standard deviations. The detection limits for FGL-1 were 0.312 ng/mL, and for sLAG-3 0.00625 ng/mL, respectively.

### Flow Cytometry

**Culture and stimulation:** PBMCs were isolated from EDTA whole blood samples using Ficoll-Hypaque (GE Healthcare Biosciences, Uppsala, Sweden) gradient centrifugation and stored at −140 °C until analysis. PBMCs were thawed and either stained immediately or stimulated. PBMCs from patients with AH and HC were stimulated in culture medium (RPMI 1640 with 100 U/mL penicillin, 100 μg/mL streptomycin and 10% heat-inactivated human AB serum) with 0.1 μg/mL anti-CD28 (Cat. No. 556620; BD Biosciences, US) in a 12-well plate pre-coated with 0.1 mg/mL anti-CD3 (Orthoclone OKT-3; Ortho Biotech, US). The cells were incubated for 48 h at 37 °C and 5% CO2. The cells were harvested in FACS running buffer (Cat. No. 130-092-747, Miltenyi Biotec, Germany).

**Staining:** Cell suspensions (5 × 106 cells/mL) were incubated with 0.1 mg/mL human immunoglobulin (Privigen, CSL Behring, US) for at least 15 min at 4 °C to reduce non-specific binding. The cells were stained with titrated volumes of fluorochrome-conjugated antibodies; CD4: PerCP-Vio 700, Cat. No. 130-113-228, (Miltenyi Biotec), CD8: PE-Vio 770, Cat. No. 130-110-680, (Miltenyi Biotec), CD25: APC, Cat. No. 170-076-079, (Miltenyi Biotec), CD69: FITC, Cat. No. 130-112-612, (Miltenyi Biotec), PD1: PE, Cat. No. 329906, (Biolegend), LAG-3: Brilliant violet 421, Cat. No. 369314, (Biolegend), Viobility: 405/520 Fixable Dye, Cat. No. 130-110-206, (Miltenyi Biotec). Incubation was carried out for 10 min at 4 °C. The cells were washed in Facs running buffer and centrifuged for 10 min at 10 °C and 240 G before analysis using a MACS Quant Analyser 10 (Miltenyi Biotec). FGL-1 staining was attempted but did not show reliable results and is not reported here.

**Gating:** FlowJo version 10.6.2 was used to analyze the data. A forward-side scatter plot was used to identify lymphocytes. Doublets were excluded. Live cells were identified by a Viobility® staining (Miltenyi Biotec). The cells were divided into CD4^+^ (T helper cells) and CD8^+^ (Cytotoxic T cells). LAG-3 expression was quantified as percentage of CD8^+^ and CD4^+^ T cells and as median fluorescence intensity (MFI). Expression of the activation markers CD25 and CD69 was measured on the LAG-3^+^ CD8^+^ and LAG-3^-^ CD8 T cells both as the percentage of positive cells and as MFI (Fig. S1). Fluorescence minus 4s (FM4s) were used as a gating control and were continuously validated by fluorescence minus one (FMOs) to ensure the validity of the FM4s.

### Statistical Analyses

The log-transformed data were analyzed for Gaussian distribution using QQ plots. The unpaired t-test was used for unpaired parametric data with only two groups. Welch t-test was used on unpaired data comparing two groups, and a Welch t-test ANOVA was used to compare more than two groups. Mann Whitney test was used on the unpaired non-parametric data comparing only 2 groups, and Kruskal Wallis analysis on the unpaired non-parametric data comparing more than 2 groups. A Wilcoxon signed-rank test was used for non-parametric paired data. If one of the compared groups was non-parametric, non-parametric analysis was applied. Due to a large intra-individual variation in the frequency of LAG-3^+^CD8^+^ T cells following stimulation, patients were divided into two groups based on cell frequencies below or above the median: LAG-3^low^ CD8^+^ and LAG-3^high^ CD8^+^ cells, respectively. Statistical analyses were performed using STATA (version 12) and GraphPad PRISM (version 8.4.3). The results are expressed as median values. Spearman's rank correlation coefficient was used to investigate correlations. Statistical significance was set at *P* < 0.05.

## Results

### Patient Characteristics

The clinical and biochemical characteristics of the patients with AH (n = 32) and stable alcohol-associated cirrhosis (n = 20) are presented in Table 1. At diagnosis of AH, the average model-of-endstage liver disease (MELD) was 22.3, and the GAHS was 8.55, which improved at day 90 of follow-up to 7.7 and 3.3, respectively. Six of the AH patients died before day 90 and further four died before the end of the 3-year follow-up. No patient underwent liver transplantation.

**Table 1:** Patient Clinical and Biochemical.

	AH Day 0 Mean (SD)	AH Day 7 Mean (SD)	AH Day 90 Mean (SD)	AC Mean (SD)
***Characteristics:***	n = 32	n = 20	n = 12	n = 20
Gender F/M	12/20	6/14	6/6	5/15
Age (years)	54.5 (11.0)	54.5 (11.2)	54.8 (11.7)	60.6 (8)^a^
ALT (U/L) [10;70]	61.2 (40.5)	84.9 (41.6)	33 (17.8)	35.6 (24)^a^
Bilirubin (μmol/L) [5;25]	256.8 (140.8)	221.3 (134)	16.5 (11.4)	23.4 (15.5)^a^
INR [<1.2]	1.81 (0.46)	1.71 (0.60)	1.28 (0.23)	1.58 (0.71)
Albumin (g/L) [36;45]	21.6 (4.6)	23.9 (4.5)	34.0 (6.5)	32.5 (5.8)^a^
CRP (mg/L) [<8]	25.3 (20.4)	18.9 (18.0)	3.2 (2.6)	4.2 (3.1)^a^
Leukocytes [3.5;10 x 10^9^/L]	11.9 (6.4)	14.1 (9.7)	6.8 (2.2)	6.8 (5.7)^a^
Neutrophils [2.7;10 x 10^9^/L]	9.25 (5.96)	11.38 (9.83)	3.74 (1.95)	
Lymphocytes [1.3;3.5 x 10^9^/L]	1.39 (0.56)	1.24 (0.37)	2.16 (0.82)	
***Complications:***				
Ascites (%)	52	35	0	
HE (%)	23	25	0	
Infection (%)	32	15	20	
***Disease severity:***				

MELD	22.30 (7.47)	21.30 (7.97)	7.67 (5.53)	11.4 (3.73)^a^
GAHS	8.55 (1.77)	8.45 (1.61)	6.33 (0.89)	
MDF	59.4 (28.1)	52.0 (37.5)	16.6 (13.0)	
Child Pugh score	11.1 (1.5)	10.7 (1.8)	7.5 (0.7)	
Treatment: Pentoxifylline/Prednisolone/Supportive care	1/15/16			
Cirrhosis on biopsy (%)	76			

AH, Alcohol-associated hepatitis; AC, alcohol-associated cirrhosis; ALT, Alanine aminotransferase; INR, international normalized ratio; CRP, C-reactive protein; HE, Hepatic encephalopathy; SD, standard deviation; MELD, Model of end-stage liver disease; GAHS, Glasgow alcoholic hepatitis score; MDF, Maddrey's Discriminant Function.

a Significant difference (*P* < 0.05) between patients with alcohol-associated hepatitis at day 0 and patients with stable alcohol-related cirrhosis.

### Liver FGL-1 Synthesis was Increased in AH Without Changes in Plasma FGL-1 Levels

**Figure 1:**
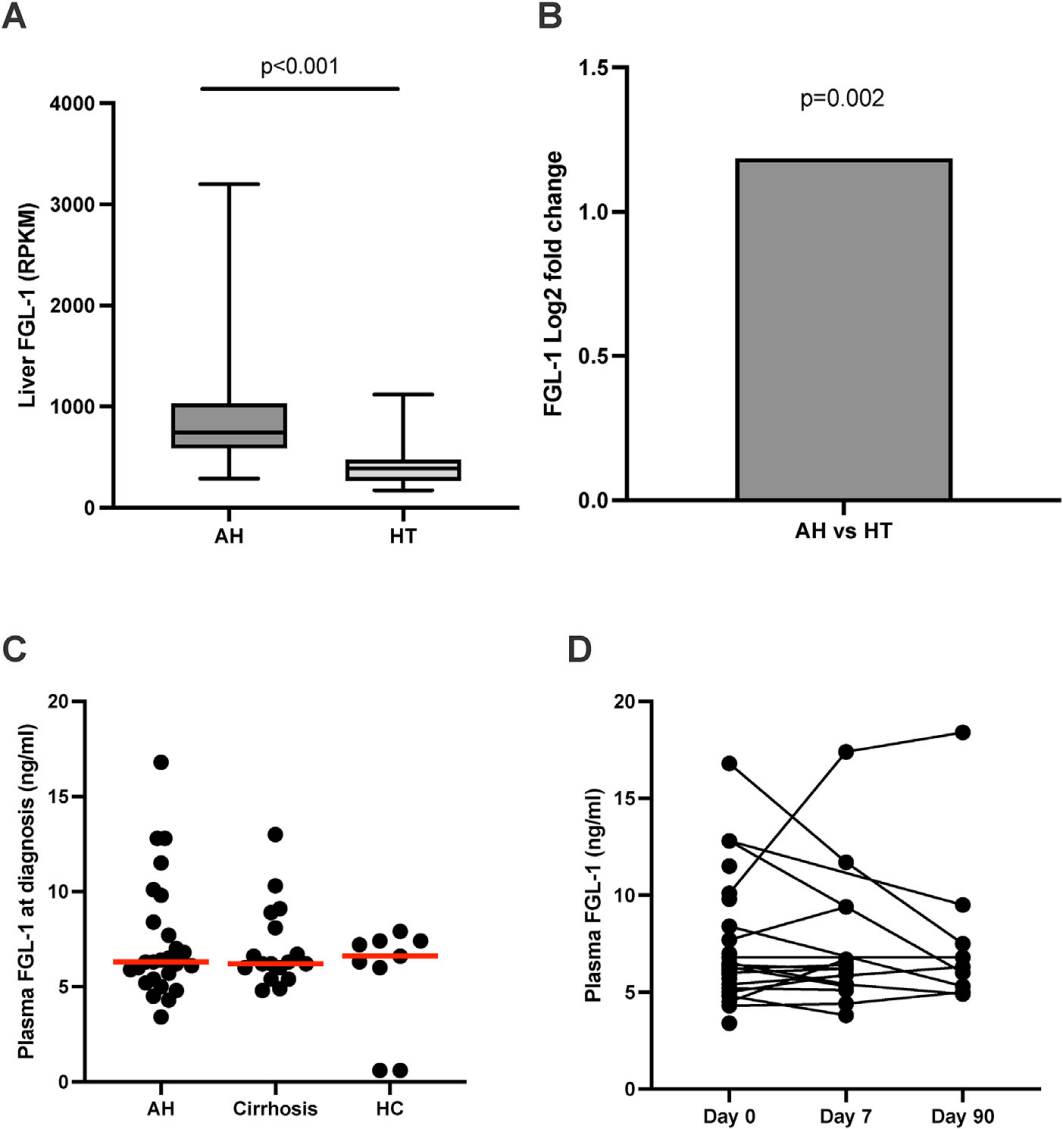
**Liver and blood fibrinogen-like protein-1 (FGL-1).** (A, B) Liver FGL-1 mRNA in patients with alcoholic hepatitis (AH) at diagnosis and healthy liver tissue (HT) measured by RNA sequencing. (C) Plasma FGL-1 in patients with AH at the time of diagnosis, patients with stable alcohol-related cirrhosis, and HC as measured by enzyme-linked immunosorbent assay (ELISA). (D) Plasma FGL-1 in the patients with AH during follow-up from day 0 to day 90 as measured by ELISA. The red line represents the median. RPKM: Reads per kilobase of transcript per million reads mapped. Differences between groups are compared using Mann–Whitney (A), One-way ANOVA (C), Mixed-effects analysis (D). The red line represents the median.

The overall liver gene expression profile was markedly different between the AH patients and the HT, and pathways involved in inflammation control were highly differentially expressed (Figure S2). Liver FGL-1 mRNA expression was increased in AH (Figure 1 A, B). There was no difference between the groups in plasma FGL-1 (Figure 1 C), and plasma FGL-1 remained stable from diagnosis until day 90 in the patients with AH (Figure 1 D). There were no differences in plasma FGL-1 levels or dynamics through follow-up between patients treated with prednisolone or not (data not shown).

### Liver LAG-3 and sLAG-3 Were Decreased and the Frequency of LAG-3^+^ T cells was Increased

**Figure 2:**
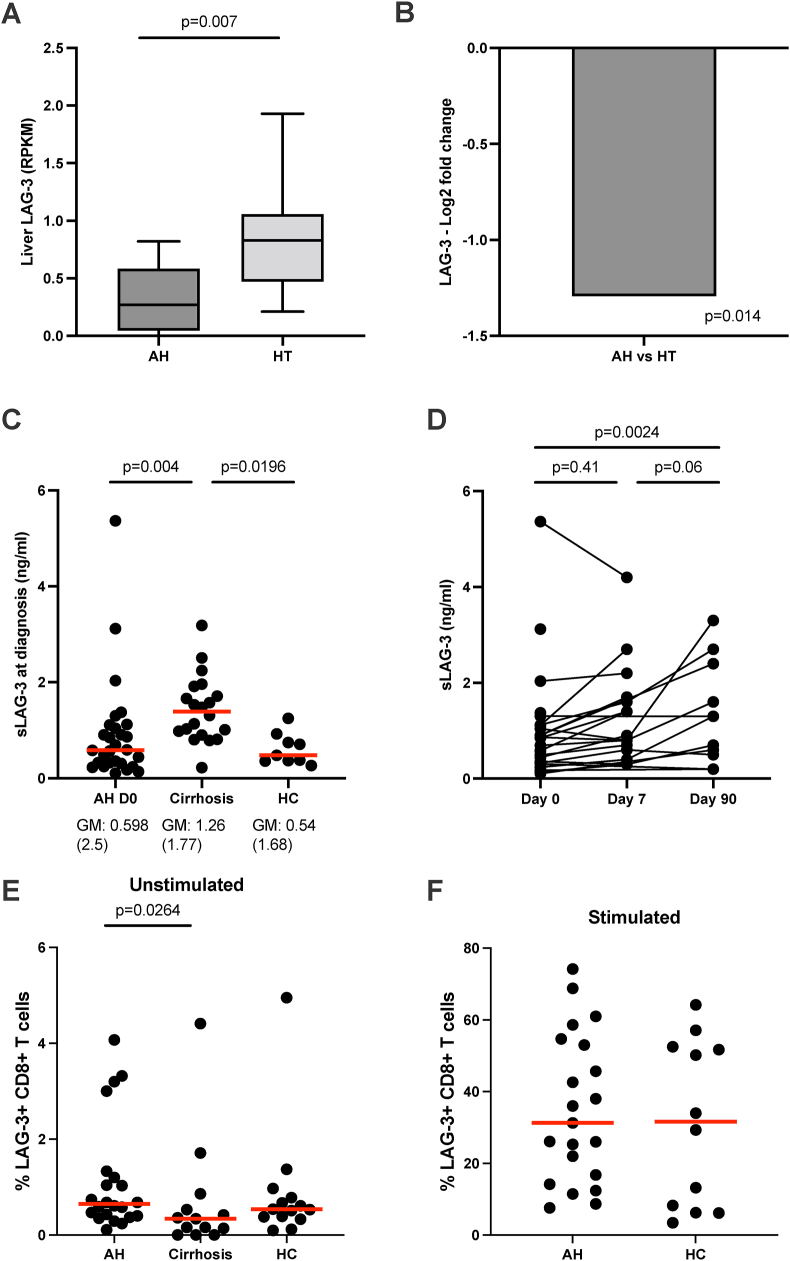
**Liver, plasma, and blood Lymphocyte Activation Gene-3 (sLAG-3).** (A, B) Liver lymphocyte Activation Gene-3 (sLAG-3) mRNA in patients with alcoholic hepatitis (AH) and healthy liver tissue (HT) measured by RNA-sequencing. (C) Plasma LAG-3 in patients with AH at diagnosis (n = 27), compared with patients with stable alcohol-related cirrhosis (n = 20) and HC (n = 9) measured by enzyme-linked immunosorbent assay measurements. (D) Plasma sLAG-3 in patients with AH during follow-up from day 0 to day 90. (D + E) Peripheral blood mononuclear cells were directly stained (E) or stimulated with anti-CD3 and anti-CD28 for 48 h prior to staining. The frequencies of LAG-3 + CD8^+^ T cells were measured by flow cytometry and compared between patients with AH at the time of diagnosis, patients with stable alcohol-related cirrhosis, and HC. Differences between groups are compared using Mann–Whitney (A), One-way ANOVA (C), Mixed-effects analysis (D), Kruskal Wallis test (E), and Mann–Whitney test (F). RPKM: Reads per kilobase of transcript per million reads mapped. The red line represents the median.

At diagnosis of AH, the expression of liver LAG-3 mRNA was decreased (Figure 2 A, B). At the same time, plasma sLAG-3 in patients was decreased to one-third of that in patients with stable alcohol-related cirrhosis (*P* = 0.004) but did not differ between AH and HC (Figure 2 C). At day 90, plasma sLAG-3 in AH was similar to that present in patients with stable alcohol-related cirrhosis and higher than in the HC (Figure 2 D). In blood, the frequency of LAG-3^+^ CD8^+^ T cells was slightly higher than in patients with stable alcohol-associated cirrhosis (*P* = 0.0264) and similar to that in HC (Figure 2 E). There was no difference in the frequencies of LAG-3^+^CD4^+^ T cells among the groups. The cells were stimulated with anti-CD3 and anti-CD28 to study the regulation of LAG-3. Following stimulation, the frequency of LAG-3^+^ CD8^+^ T cells increased 30-fold, equally so in AH and HC (Figure 2 F). Liver LAG-3 mRNA expression was strongly associated with the frequency of both unstimulated and stimulated LAG-3^+^ CD8^+^ T cells (r = 0.87, *P* = 0.024 and r = 0.93, *P* = 0.008, respectively), but not the frequency of LAG-3^+^ CD4^+^ T cells. These findings were not different from patients treated with prednisolone or not (data not shown).

### The LAG-3^+^CD8^+^ T cells Expressed Markers of Activation

To understand whether LAG-3 upregulation was associated with cell activation status, we measured the expression of CD25 and CD69 following stimulation. The expression of the activation markers CD25 and CD69 were higher on the LAG-3^+^ than the LAG-3^-^ CD8^+^ T cells in patients with AH and HC suggesting that they represent a subgroup of highly activated T cells (Figure S2). The expressions of CD25 and CD69 on the LAG-3^+^ CD8^+^ T cells were high and similar in patients with AH and HC. This was the case both when measuring the frequency of positive cells and the MFI (Figure S2).

### FGL-1 at Diagnosis was Associated With High Disease Severity and High Mortality

**Figure 3:**
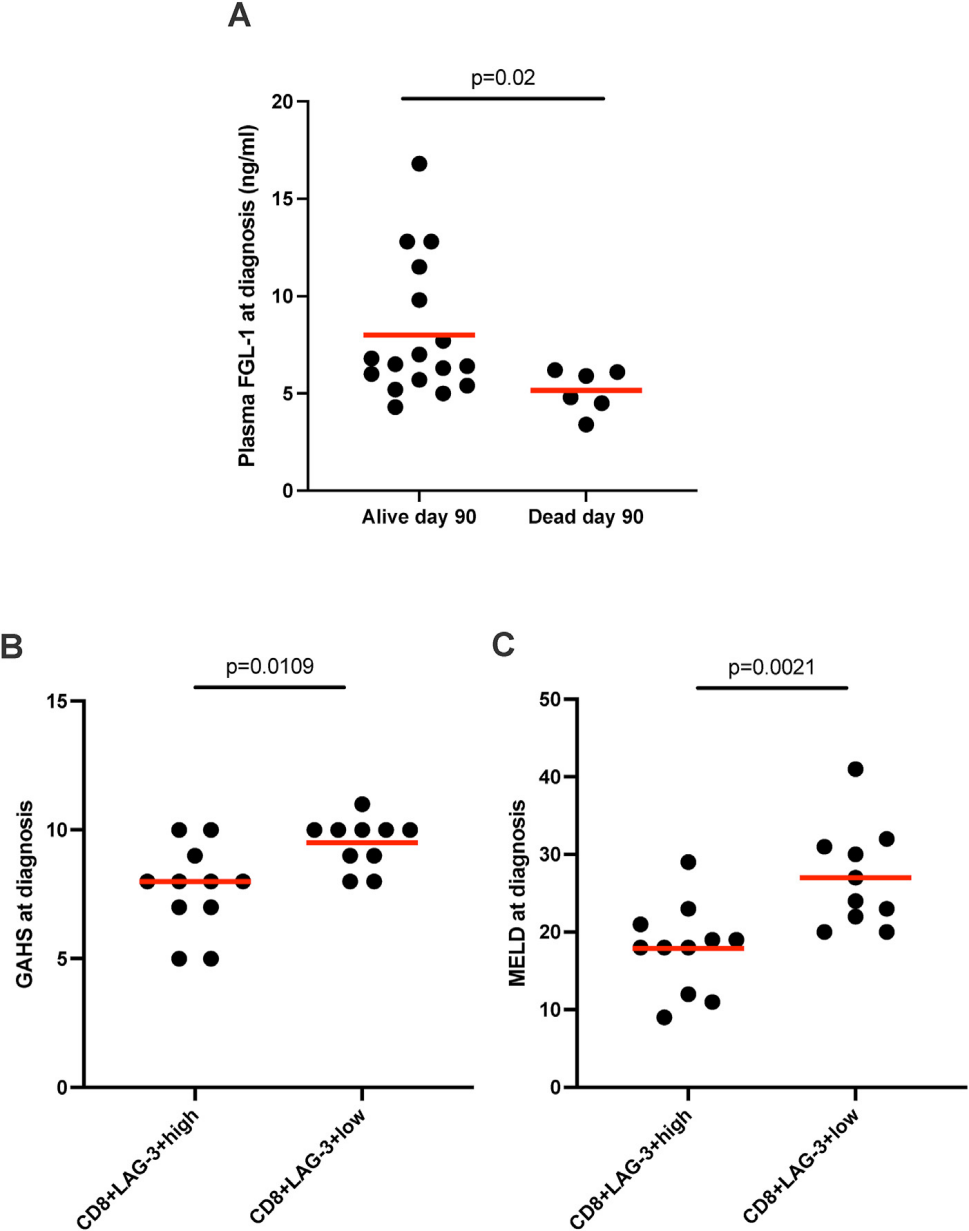
**An adverse outcome in patients with low plasma FGL-1 and low frequency of LAG-3**^**+**^**CD8**^**+**^**T cells at diagnosis.** (A) Plasma FGL-1 measured by ELISA at diagnosis in patients with AH. The patients are divided into those who were alive at day 90 and those who had died before or at day 90 after diagnosis. (B) Glasgow Alcoholic Hepatitis Score (GAHS), and (C) Model of End-stage Liver Disease (MELD) score compared between patients with alcoholic hepatitis (AH) who were divided into two groups based on the frequency of LAG-3^+^CD8^+^ T cells at diagnosis as measured by flow cytometry: above the median (LAG3^+^_high_ CD8^+^) or below the median (LAG-3^+^_low_ CD8^+^). Differences between groups are compared using Mann Whitney test (A–C). The red line represents the median.

We then examined whether these findings were related to the clinical disease severity and course. Patients with AH and a low level of plasma FGL-1 at diagnosis had a more severe disease as measured by a higher Child-Pugh score (r = −0.54, *P* = 0.02) and a lower PP (coagulation factors II, VII, X) (r = 0.51, *P* = 0.01). Likewise, the six patients who died before day 90, and the total of 10 patients who died within three years had a lower plasma FGL-1 at diagnosis than those who were still alive (*P* = 0.02, *P* = 0.03, respectively) (Figure 3 A). There was also a tendency for lower liver FGL-1 mRNA expression in patients who had died within three years (*P* = 0.087). There were no associations between FGL-1, MELD, and GAHS (Supplementary Table 1).

### LAG-3 at Diagnosis was Associated With High Disease Severity

Low liver LAG-3 mRNA levels at diagnosis were correlated with higher MELD (r = −0.55, *P* = 0.05) and Child-Pugh scores (r = −0.66, *P* = 0.02). In addition, when we divided the patients with AH into two groups based on whether their frequency of LAG-3^+^ CD8^+^ T cells was below (LAG-3^low^ CD8^+^) or above (LAG-3^high^ CD8^+^) the median, the LAG-3^low^ CD8^+^ patients had more severe disease than the LAG-3^high^ CD8^+^ patients (Figure 3 B, C). There were no associations between plasma sLAG-3 levels at diagnosis and disease severity scores or death by day 90.

## Discussion

This study provides support for the implication of the FGL-1/LAG-3 axis in AH pathogenesis and disease course. We measured increased liver FGL-1 and decreased liver LAG-3 expression at diagnosis of AH and found that the patients with the lowest plasma FGL-1 together with low liver LAG-3 had higher disease severity and mortality. We found the same for the patients with a low frequency of LAG-3^+^ CD8^+^ T cells. Overall, these findings may support the hypothesis that the loss of intact local paracrine regenerative effects, along with weakened control of T cell-driven inflammation, contributes to the negative outcome of AH.

The increase in liver FGL-1 mRNA expression aligns with what is reported in a rodent model of acute liver injury.^22^ In agreement with that study, our data indicate a harmful effect of low FGL-1 in the acute liver injury of AH. This finding is consistent with previous experimental studies, which have explored the possible mechanisms underlying the protective effect of FGL-1. FGL-1 has been shown to protect against endoplasmic reticulum stress.^10^ Also, FGL-1 was shown to reduce apoptosis of hepatocytes, increase their proliferation, and reduce the production of pro-inflammatory cytokines, thus protecting against acute liver injury.^10^^,^^23^^,^^24^ There are likely other paracrine mechanisms of action in play in the liver such as modulation of various metabolic pathways, which have also been described in rodent models.^17^^,^^25^

FGL-1 is produced by hepatocytes and is known to be strongly upregulated during inflammation and liver regeneration, and therefore can be classified as an acute phase protein.^8^^,^^10^^,^^22^ The relative reduction of FGL-1 observed in some AH patients may reflect the liver's synthetic dysfunction during AH, as is known from clinical experience with C-reactive protein, for example. Low FGL-1 may compromise the liver's ability to regenerate, which is known to be associated with poor prognosis.^12^ Lower plasma FGL-1 at diagnosis was associated with increasing Child-Pugh Score, but this association, albeit in a similar direction (low FGL-1, higher disease severity), did not reach statistical significance using MELD as a disease severity measure. A possible but speculative explanation is that the Child-Pugh score more accurately captures features of acute disease deterioration in AH, such as ascites and hepatic encephalopathy, and therefore is associated with the acute phase protein FGL-1.

FGL-1 is a ligand for LAG-3. The relative lowering of FGL-1 in AH may lead to decreased inflammation control and contribute to disease deterioration, due to loss of FGL-1 - LAG-3 interactions.^13^^,^^26^ This may be further worsened by the lowering of LAG-3 in AH which we detected in liver biopsies, and also by the relative lowering of plasma sLAG-3 comparing levels at diagnosis to higher levels at day 90. Our data suggest that plasma sLAG-3 is depressed from a habitual higher level as part of the acute episode of AH. Although this level was not different from that of HC, we saw an increase in sLAG-3 through the 90-day follow-up, which reached the elevated sLAG-3 levels observed in the patients with stable alcohol-associated cirrhosis. These cirrhosis patients were included as sick controls because the AH patients in the majority of cases had underlying liver cirrhosis.

The large variation in the LAG-3^+^CD8^+^ T cells' response to stimulation *ex vivo* is in line with previous studies.^27^ So is the lowering of plasma sLAG-3 compared to patients with stable alcohol-associated cirrhosis.^28^ As LAG-3 is among the T cell inhibitory receptors whose expression may reflect T cell exhaustion, we stimulated the cells to examine whether they were functionally inert. We showed that the LAG-3^+^CD8^+^ T cells can upregulate both CD25 and CD69- like cells from HC, indicating that these cells are functionally active. Of note, LAG-3 was only regulated on CD8^+^ T cells in our patients with AH, and only the CD8^+^ LAG-3 expression was associated with liver LAG-3 mRNA levels suggesting that this checkpoint inhibitor may be particularly important for CD8^+^ T cells in AH.

It is a strength of the study that the patient cohort and its clinical course are well-characterized, followed longitudinally, and include both liver and blood samples. However, there are several limitations to the study. The study is descriptive, and the assays are done *ex vivo*. Therefore, we can not answer whether the loss of inhibitory LAG-3 or its ligand FGL-1 is a cause or consequence of the disease. Further, we studied a relatively small single-centerpatient group, which also limits the precision of our interpretations. The clinical associations we describe all point in the same direction but do not all reach statistical significance. The small transjugular liver biopsies did not procure sufficient tissue to perform protein measurements or qPCR to back our mRNA measurements. The sum of these limitations implies that our study should be considered an early report on the possible significance of the FGL-1-LAG-3 axis in AH that presents findings motivating further exploration.

In conclusion, this study demonstrates elevated FGL-1 expression in the liver in AH, and at the same time lowered LAG-3 expression in liver and blood. The patients with low FGL-1 and low LAG-3 fared worst. The possible involvement of the FGL-1-LAG-3 axis in AH may point towards new aspects of the hepatocyte regenerative defects and the uncontrolled inflammation that are associated with severe presentation and course of AH.

## CRedIT authorship contribution statement

Conceptualization, Frederik Brix, Bent Deleuran, Thomas Sandahl and Sidsel Støy; Formal analysis, Lasse Pedersen, Lotte Eriksen and Sidsel Støy; Methodology, Lasse Pedersen, Lotte Eriksen, Frederik Brix and Sidsel Støy; Supervision, Hendrik Vilstrup, Bent Deleuran, Thomas Sandahl and Sidsel Støy; Writing – original draft, Lasse Pedersen, Lotte Eriksen and Sidsel Støy; Writing – review & editing, Hendrik Vilstrup, Bent Deleuran, Thomas Sandahl and Sidsel Støy.

## Funding

The study was supported by grants from the *Novo Nordisk Foundation* and *Aase and Ejnar Danielsens Fond*.

## Declaration of competing interest

The authors have no conflicts of interest to declare.

## References

[1] Lucey M.R., Mathurin P., Morgan T.R. (2009). Alcoholic hepatitis. N Engl J Med.

[2] Sandahl T.D., Jepsen P., Thomsen K.L., Vilstrup H. (2011). Incidence and mortality of alcoholic hepatitis in Denmark 1999-2008: a nationwide population based cohort study. J Hepatol.

[3] Liu S.Y., Tsai I.T., Hsu Y.C. (2021). Alcohol-related liver disease: basic mechanisms and clinical perspectives. Int J Mol Sci.

[4] Szabo G. (2015). Gut–liver Axis in alcoholic liver disease. Gastroenterology.

[5] Singal A.K., Shah V.H. (2019). Current trials and novel therapeutic targets for alcoholic hepatitis. J Hepatol.

[6] Bajaj J.S. (2019). Alcohol, liver disease and the gut microbiota. Nat Rev Gastroenterol Hepatol.

[7] Lange C.M.A.-J., Rawitzer J., Willuweit K., Canbay A., Baba H.A. (2022). Cirrhosis-based acute-on-chronic liver failure is marked by inflammation and impaired liver regeneration despite Stat3 activation. Gastro Hep Advances.

[8] Liu Z., Ukomadu C. (2008). Fibrinogen-like protein 1, a hepatocyte derived protein is an acute phase reactant. Biochem Biophys Res Commun.

[9] Yu H.T., Yu M., Li C.Y. (2009). Specific expression and regulation of hepassocin in the liver and down-regulation of the correlation of HNF1alpha with decreased levels of hepassocin in human hepatocellular carcinoma. J Biol Chem.

[10] Gao M., Zhan Y.-Q., Yu M. (2014). Hepassocin activates the EGFR/ERK cascade and induces proliferation of L02 cells through the Src-dependent pathway. Cell Signal.

[11] Li C.Y., Cao C.Z., Xu W.X. (2010). Recombinant human hepassocin stimulates proliferation of hepatocytes in vivo and improves survival in rats with fulminant hepatic failure. Gut.

[12] Lanthier N., Rubbia-Brandt L., Lin-Marq N. (2015). Hepatic cell proliferation plays a pivotal role in the prognosis of alcoholic hepatitis. J Hepatol.

[13] Wang J., Sanmamed M.F., Datar I. (2019). Fibrinogen-like protein 1 is a major immune inhibitory ligand of LAG-3. Cell.

[14] Andrews L.P., Marciscano A.E., Drake C.G., Vignali D.A. (2017). LAG3 (CD223) as a cancer immunotherapy target. Immunol Rev.

[15] Anderson A.C., Joller N., Kuchroo V.K. (2016). Lag-3, tim-3, and TIGIT: Co-inhibitory receptors with specialized functions in immune regulation. Immunity.

[16] Goldberg M.V., Drake C.G. (2011). LAG-3 in cancer immunotherapy. Curr Top Microbiol Immunol.

[17] Wu H.T., Lu F.H., Ou H.Y. (2013). The role of hepassocin in the development of non-alcoholic fatty liver disease. J Hepatol.

[18] Maruhashi T., Okazaki I.M., Sugiura D. (2018). LAG-3 inhibits the activation of CD4(+) T cells that recognize stable pMHCII through its conformation-dependent recognition of pMHCII. Nat Immunol.

[19] Jerrells T.R. (2002). Role of activated CD8+ T cells in the initiation and continuation of hepatic damage. Alcohol.

[20] Stoy S., Dige A., Sandahl T.D. (2015). Cytotoxic T lymphocytes and natural killer cells display impaired cytotoxic functions and reduced activation in patients with alcoholic hepatitis. Am J Physiol Gastrointest Liver Physiol.

[21] Støy S., Laursen T., Glavind E. (2020). Low interleukin-22 binding protein is associated with high mortality in alcoholic hepatitis and modulates interleukin-22 receptor expression. Clin Transl Gastroenterol.

[22] Hara H., Uchida S., Yoshimura H. (2000). Isolation and characterization of a novel liver-specific gene, hepassocin, upregulated during liver regeneration. Biochim Biophys Acta Gene Struct Expr.

[23] Yang Y., Zhai H., Wan Y. (2021). Recombinant human HPS protects mice and nonhuman primates from acute liver injury. Int J Mol Sci.

[24] Yang Y., Chen H., Wan Y. (2022). Protective role of hepassocin against hepatic endoplasmic reticulum stress in mice. Int J Mol Sci.

[25] Demchev V., Malana G., Vangala D. (2013). Targeted deletion of fibrinogen like protein 1 reveals a novel role in energy substrate utilization. PLoS One.

[26] Graydon C.G., Mohideen S., Fowke K.R. (2020). LAG3's enigmatic mechanism of action. Front Immunol.

[27] Zelba H., Bedke J., Hennenlotter J. (2019). PD-1 and LAG-3 dominate checkpoint receptor–mediated T-cell inhibition in renal cell carcinoma. Cancer Immunol Res.

[28] Riva A., Palma E., Devshi D. (2021). Soluble TIM3 and its ligands galectin-9 and CEACAM1 are in disequilibrium during alcohol-related liver disease and promote impairment of anti-bacterial immunity. Front Physiol.

