# Supplementary material for: The FGL-1/LAG-3 Axis is Associated With Disease Course in Alcohol-associated Hepatitis: A Preliminary Report
Source: J Clin Exp Hepatol. 2024 Oct 10;15(1):102424. doi: 10.1016/j.jceh.2024.102424 (PMC11567029; doi:10.1016/j.jceh.2024.102424)
Supplement: Multimedia component 1 [file mmc1.docx]

**Supplementary material**

**Manuscript title:** The FGL-1/LAG-3 axis is associated with disease course in alcoholic hepatitis

**Authors:** Lasse Pedersen, Lotte Lindgreen Eriksen, Frederik Heiberg Brix, Hendrik Vilstrup, Bent Deleuran, Thomas Damgaard Sandahl, Sidsel Støy.

**Table of contents**

Supplementary Figure 1

Supplementary Figure 2

Supplementary Figure 3

Supplementary Table 1

***
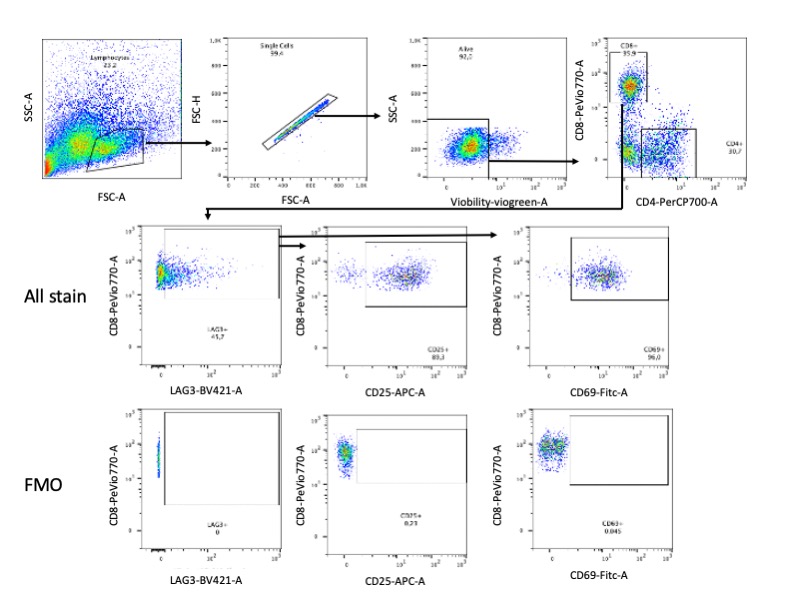
***

**Supplementary Figure 1. Gating strategy.**

The lymphocyte population is defined on a forward-side scatter plot (FSC-SSC) and duplets are then excluded. Live cells are identified as Viobility® negative cells, which are then divided into CD4 and CD8 positive T cells. The percentage of LAG-3 positive, CD25 positive and CD69 positive events were determined based on an fluorescence minus one control.

**Supplementary Figure 2. Summary presentation of RNA sequencing data.**

A. Principal component analysis of the 500 most variable genes. The points indicate the relationship between individuals across their gene expression profiles. Values in brackets indicate the amount of variability in percent explained by the axis. B. Visualisation of the regulation of different pathways in the patients with alcohol-associated hepatitis (AH) compared with the healthy controls (HC). Pathway perturbation is represented as the number of differentially expressed genes in the pathway, divided by the total number of genes in the pathway.


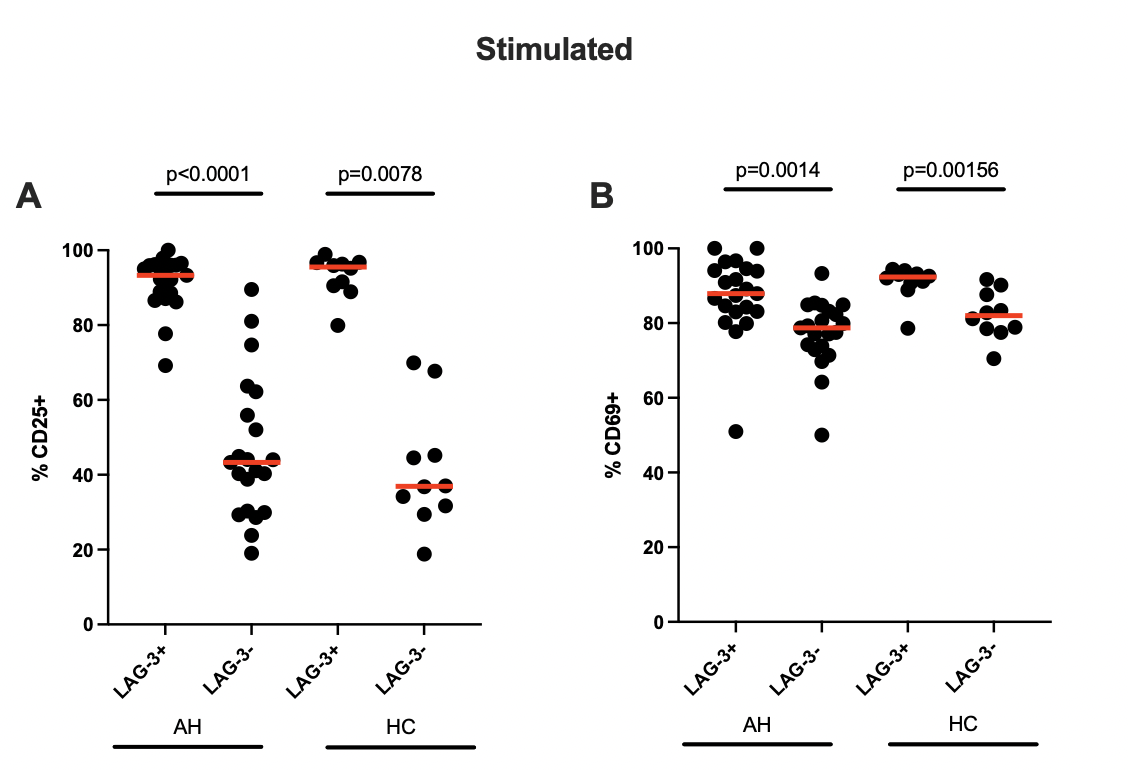


**Supplementary Figure 3. LAG-3^+^ T cells express CD25 and CD69.**
Peripheral blood mononuclear cells were stimulated for 48 hours with anti-CD3 and anti-CD28. The percentage of Lymphocyte Activation Gene-3 (LAG-3) positive (+) and negative (-) CD8 ^+^ T cells positive for the activation markers CD25 (A/C) and CD69 (B/D) are reported in patients with alcoholic hepatitis (AH) at the time of diagnosis and healthy controls (HC), measured using flow cytometry. Differences between groups are compared using a Wilcoxon Signed Rank test (A+B).

**Supplementary Table 1. Overview of correlations.**

| **Correlations:** | **Liver FGL-1 mRNA** | **FGL-1 d0** | **Liver LAG-3 mRNA** | **sLAG3 d0** |
| --- | --- | --- | --- | --- |
| MELD | r = - 0.02  p = 0.94 | r = - 0.27  p = 0.21 | **r = - 0.55 p = 0.05** | r = 0.24  p = 0.25 |
| GAHS | r =- 0.3  p = 0.31 | r = - 0.21  p = 0.32 | r = - 0.29  p = 0.33 | r = 0.12  p = 0.57 |
| Child Pugh | r = - 0.05  p = 0.86 | **r = - 0.52**  **p = 0.01** | **r = - 0.66**  **p = 0.02** | r = 0.33  p = 0.1 |
| PP | r = - 2.2  p = 0.46 | **r = 0.51**  **p = 0.01** | r = 0.28  p = 0.36 | **r = - 0.41**  **p = 0.037** |
| Bilirubin | r = - 0.32  p = 0.29 | r = - 0.06  p = 0.79 | r = - 0.43  p = 0.14 | r = -0.02  p = 0.92 |
| INR | r = 0.29  p = 0.34 | **r = - 0.48**  **p = 0.02** | r = - 0.26  p = 0.38 | **r = 0.43**  **p = 0.03** |

*Correlations between FGL-1 and LAG-3 levels in liver and plasma in patients with alcoholic hepatitis at diagnosis and disease severity scores and biochemical characteristics at diagnosis. Statistically significant correlations (p<0.05) are highlighted. All correlations were done using the Spearman’s rank correlation coefficient.* MELD: Model of End-stage Liver disease. GAHS: Glasgow Alcoholic Hepatitis Score.
